# Analysis of pole acceleration in spatial motions by the generalization of pole changing velocity

**DOI:** 10.1007/s00707-019-02408-9

**Published:** 2019-05-09

**Authors:** Gábor Csernák

**Affiliations:** 1 Department of Applied Mechanics, Budapest University of Technology and Economics, Budapest, Hungary; 2 MTA-BME Research Group on Dynamics of Machines and Vehicles, Budapest, Hungary

## Abstract

It is well known in planar kinematics of rigid bodies that the acceleration of the material point coinciding with the instantaneous center of rotation (or pole) is perpendicular to the so-called pole changing velocity. In the present paper, the concept of pole changing velocity is generalized to spatial motions. Using this result, the acceleration of the material points along the instantaneous screw axis can be expressed in a straightforward way, without the tools of advanced differential geometry.

## Introduction

Rigid body kinematics is a subject that belongs partly to geometry, partly to dynamics. Accordingly, there are approaches to this branch of science that are based on purely geometrical derivations [1–3]. Other authors—especially in books written for mechanical engineers [4–6]—put more emphasis on the time-based treatment of the important concepts, i.e., the main results are derived and formulated by using position, velocity and acceleration vectors. Geometrical kinematics has several advantages over the time-based approach in terms of mathematical elegance and rigor [7]. However, the learning (and teaching) of geometrical methods requires the application of advanced mathematical tools. Since university curricula rarely provide the necessary time to properly cover these topics, only specialists can apply geometrical kinematics to the solution of practical problems—this is why the use of time-based methods can still be justified.

The present paper focuses on the pole changing velocity that characterizes the change of the geometric position of the instantaneous center of rotation (or pole) in the case of planar motions and nonzero angular velocity. The goal of this contribution is to show that the concept of pole changing velocity can be generalized to spatial motions by using Euler’s rigid body formulas and exploiting that all motions can be interpreted as rolling or sliding of two ruled surfaces on each other. On the basis of this generalization, the acceleration of the material points along the instantaneous screw axis (ISA) can be expressed in a straightforward way, without the tools of advanced differential geometry. The obtained results may help to visualize the connection between the velocity distribution and the acceleration distribution during the spatial motion of a rigid body.

### Background and literature survey

**Fig. 1:**
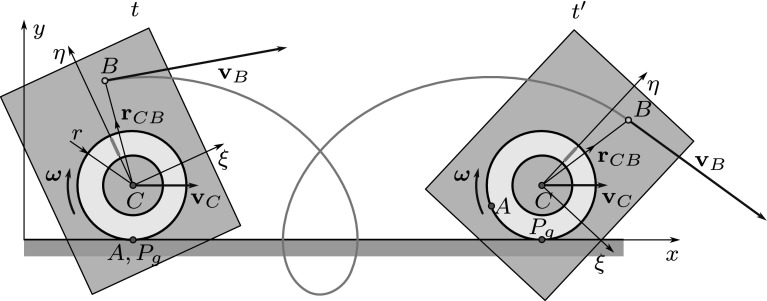
Planar rolling of a ring-shaped body on a horizontal path. The extension of the body is illustrated by a shaded rectangle. The velocity of points *C* (center of gravity) and *B* can be defined only by the extension of the body since both points are situated outside the contours of the ring

The analysis of rigid body motion can often be facilitated if special points—e.g., points with zero velocity or acceleration—can be found based on simple geometric concepts. In many cases, these special points are outside the contours of the body. To solve this problem, usually an extended body is considered by imagining that nearby points move together with the real rigid body (Fig. 1).

**Fig. 2:**
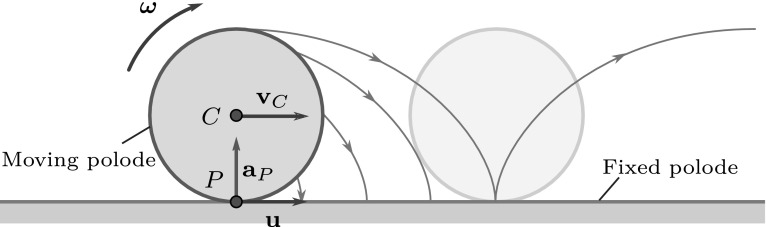
Moving and fixed polodes (centrodes) of a rolling wheel exhibiting planar motion. The pole changing velocity $\mathbf {u}$ and the pole acceleration $\mathbf {a}_P$ are also shown

Throughout the paper, each reference to the material points of the rigid body should be interpreted based on this extension. For example, the velocity and acceleration of point *B* can be expressed in this sense using Euler’s rigid body formulas:

1 $$\begin{aligned} \mathbf {v}_B&= \mathbf {v}_C + \varvec{\omega }\times \mathbf {r}_{CB}, \end{aligned}$$

2 $$\begin{aligned} \mathbf {a}_B&= \mathbf {a}_C + \varvec{\alpha }\times \mathbf {r}_{CB} + \varvec{\omega }\times (\varvec{\omega }\times \mathbf {r}_{CB}), \end{aligned}$$

where $\mathbf {v}_B$, $\mathbf {v}_C$ and $\mathbf {a}_B$, $\mathbf {a}_C$ denote the velocities and accelerations of the corresponding points, respectively. $\mathbf {r}_{CB}$ is the position vector, $\varvec{\omega }$ is the angular velocity and $\varvec{\alpha }= {\dot{\varvec{\omega }}}$ denotes the angular acceleration of the body.

In the case of planar motion of rigid bodies, there exists a point on the (extended) body that is instantaneously at rest, provided that the angular velocity $\varvec{\omega }$ of the body is nonzero. This point has several names in the literature. It is referred to as instant center of velocity [4, 5], instant center [8], instantaneous center of rotation [9], velocity pole [1] or simply, pole [10, 11]. For the sake of simplicity, we adopt the latter denomination. Note that certain authors [4] use the term “pole” for finite displacements, while the corresponding point for infinitesimal displacements is called “instantaneous center of velocity.”

The notion of pole was introduced by Johann Bernoulli [12] in the eighteenth century, for the characterization of planar motions. As the body moves, the geometric position of the pole changes continuously (Fig. 2). To describe this phenomenon, Poinsot [13] introduced the notions of moving and fixed polodes [1] or centrodes [9]. These curves (also referred to as body curve and space curve) describe the earlier and future geometric positions of the pole in the moving reference frame of the rigid body and in the fixed reference frame, respectively. During the motion, the moving polode rolls on the fixed polode without slip. In each instant, the actual contact point of these curves defines the pole.

In order to avoid any misunderstanding, it is worth to distinguish between two different interpretations of the pole. On the one hand, the pole can be thought of as a geometric object [2], defined by the requirement that the velocity of the material point of the body coinciding with it is zero. The term *geometric pole* with the notation $P_g$ will be used throughout this paper when talking about the pole in this sense. On the other hand, the pole can be considered as a material point of the body in the examined time instant *t*, that has zero velocity. As follows, the pole in this “material” sense will be simply referred to as *pole* and denoted by *P*.

The difference between the geometric pole and the pole is illustrated in Fig. 1, where the moving polode is the outer contour of the ring, while the fixed polode is found on the surface of the ground. The letter *A* denotes a material point of the body that coincides with the geometric pole $P_g$ (the contact point of polodes) at time *t*; thus, $A \equiv P$ is the pole in the subfigure on the left. These two points are located in different places at a later time instant $t'$, so point *A* is not a pole anymore in the subfigure on the right.

The geometric pole and the pole coincide in each time instant, but their velocity and acceleration are typically different. Although the velocity of the pole (as a material point) is zero, the geometric pole (that coincides with another material point in a subsequent time instant) apparently moves along the fixed polode. In the examples shown in Figs. 1 and 2, the pole is always below the center of gravity; thus, the velocity of this apparent motion—denoted by $\mathbf {u}$—is equal to the velocity of the center of gravity $\mathbf {v}_C$.

There is no generally accepted name for the velocity characterizing the rate of change of the position of the geometric pole. We will use the term *pole changing velocity*, according to [10, 11]. This physical quantity is referred to as pole velocity in [1], IC velocity in [4], instant center’s velocity in [5] and pole transfer velocity in [14]. Other authors only paraphrase the velocity of the geometric pole without assigning a name to it, for example: “geometric velocity at which the contact changes along the centrodes” [2], “displacement velocity of the instantaneous center” [4], “evolution velocity of instant center of rotation,” “speed of change” or “velocity vector” of the instant center of rotation [15], “the velocity with which the instant center propagates along the outline of the body” [16], or “speed of progression of the rolling point along the centrode [17].”

It is well known in the planar case that the pole changing velocity $\mathbf {u}$ is parallel to the common tangent of the fixed polode and the moving polode [1]. Moreover—since the path of the pole has a cusp at the contact point of the two polodes, as shown in Fig. 2—the acceleration $\mathbf {a}_P$ of the pole (the material point) is just perpendicular to the pole changing velocity $\mathbf {u}$ [2, 5, 9] and $|\mathbf {u}| = |\mathbf {a}_P/\omega |$ [18].

**Fig. 3:**
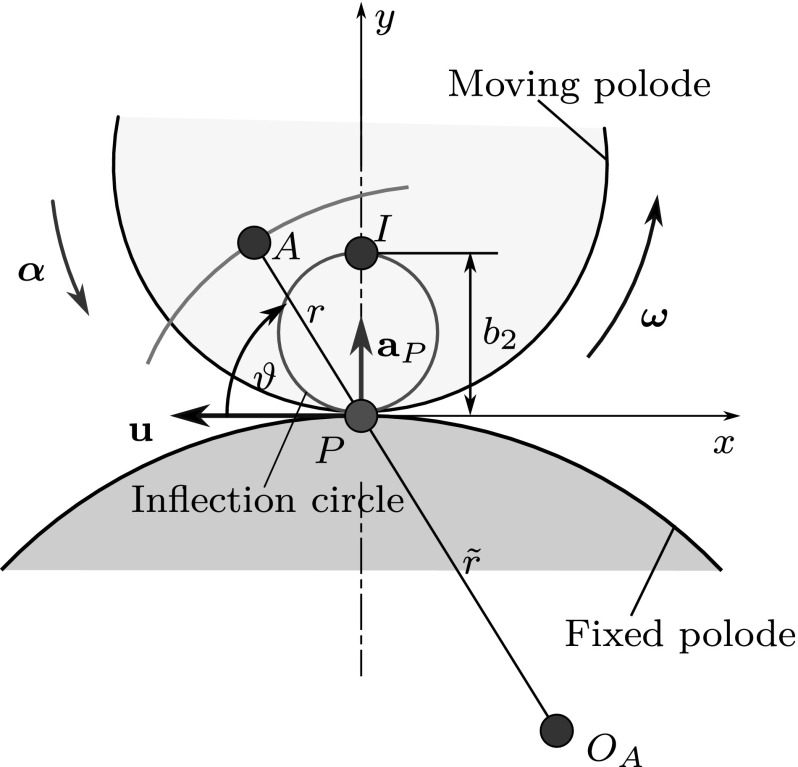
Illustration of the Euler–Savary theorem that establishes a connection between the distances $r\equiv {\overline{AP}}$, ${\tilde{r}} \equiv \overline{O_A P}$ and the diameter $b_2$ of the inflection circle (red). $O_A$ is the center of curvature of the path of point *A*. The moving and fixed polodes, a section of the path of point *A* (blue), the pole changing velocity $\mathbf {u}$ and the pole acceleration $\mathbf {a}_P$ are also shown. *I* denotes the so-called inflection pole with acceleration $\mathbf {a}_I \perp \mathbf {r}_{IP}$ (color figure online)

Although the author did not find a complete proof in the literature, the spatial generalization of the connection between $\mathbf {u}$ and $\mathbf {a}_P$ can be derived based on the Euler–Savary theorem. The original, planar version of this theorem establishes the connection between the positions of three collinear points: a point *A* on the rigid body, the pole *P* and the center of curvature $O_A$ of the path of point *A*. Using a polar coordinate system with the origin at the pole *P*, one obtains [2, 4]

3 $$\begin{aligned} \left( \frac{1}{r}-\frac{1}{{\tilde{r}}}\right) \sin (\vartheta ) = \frac{1}{b_2}, \end{aligned}$$

where the positions of *A* and $O_A$ are $(r,\vartheta )$ and $({\tilde{r}},\vartheta )$, respectively, while $b_2$ is the diameter of the so-called inflection circle, as shown in Fig. 3. The inflection circle is the locus of points with zero normal acceleration.

It is shown in [16] that the diameter of the inflection circle can be expressed by the pole changing velocity and the magnitude of the angular velocity $\varvec{\omega }$ of the rigid body: $|b_2| = |\mathbf {u}/\omega |$. Moreover, the magnitude of the acceleration of the pole can be expressed as $|\mathbf {a}_P| = |b_2| \omega ^2$ [9]. This latter result follows trivially from Euler’s acceleration formula if one utilizes that $\mathbf {a}_P \perp \mathbf {a}_I$, where *I* denotes a point on the inflection circle, the so-called inflection pole (also shown in Fig. 3).

Most of the aforementioned results have been generalized to spatial or spherical motions. Mozzi [19] and Chasles [20] introduced the so-called screw axis and formulated the following theorem: Each Euclidean displacement in three-dimensional space has a screw axis, and the displacement can be decomposed into a rotation about and a slide along this screw axis. The spatial motion of a body can be considered as a continuous set of displacements. Applying Chasles’ theorem to infinitesimally small displacements, a well-defined screw axis—the instantaneous screw axis (ISA)—can be assigned to the rigid body at any time instant.

**Fig. 4:**
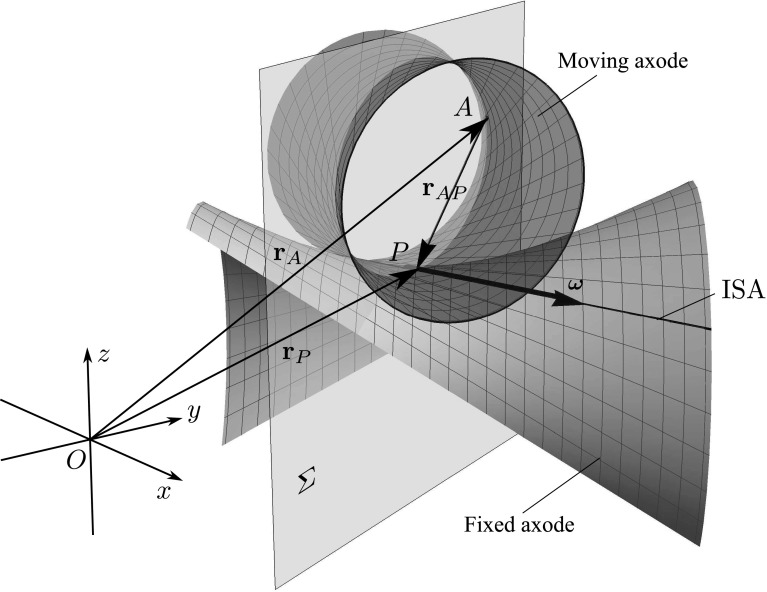
Example of spatial motion: raccording motion of a hyperboloid on a fixed hyperboloid. The ISA is a common generator of the two ruled surfaces. The velocity of point *P* is zero or parallel with the ISA. Point *A* is an arbitrarily chosen point of the moving body such that plane $\varSigma$—that is perpendicular to the angular velocity $\varvec{\omega }$ of the moving body—passes through both *A* and *P*

During the continuous motion of the body, the ISA generates two ruled surfaces: the *fixed axode* in the fixed reference frame (corresponding to the fixed polode in the planar case) and the *moving axode* (counterpart of moving polode) in the body-fixed frame. It can be shown [9] that at every point of the common ISA the tangent planes of these surfaces coincide. As a consequence, the most general type of continuous motion is the so-called raccording motion, that is, the translation along the ISA and a rotation about the ISA. An example of such a motion is illustrated in Fig. 4.

The Euler–Savary theorem had been generalized to the spatial case by Distelli [21, 22], and it was expressed by the pole changing velocity in the spherical case in [10, 11]. The concept of inflection circle was also generalized to spherical motions: The inflection cone (points with zero normal acceleration) and normal cone (points with zero tangential acceleration) are introduced in [2]. Based on these geometric results, the connections between the pole changing velocity and the acceleration of the points along the ISA can be established.

Despite its geometric nature, there are still several unsolved problems in kinematics. Some of the latest results are practice oriented [23], others are more inclined toward the pure theoretical extension of known concepts [24], or the goal is the determination of all possible motion types if the displacement of the body is not completely specified [25].

### Formulation of the problem of interest and the scope of this study

As was mentioned in the previous section, the connections between the velocities and accelerations of spatially moving rigid bodies are established in principle. However, the goal of the books and papers cited in the previous section is the general geometric description of the motion properties. Consequently, advanced tools of differential geometry are used in them. Although this approach is elegant and powerful, the complexity of the mathematical tools may discourage potential readers from the application of the results. Moreover, as the literature review of the previous section shows, there are no generally accepted terms for the pole and the pole changing velocity. The diversity of the used terminology also hinders the orientation of engineers in this field.

Certain authors made successful attempts to derive many of the aforementioned results using time-based concepts—position, velocity and acceleration vectors—while keeping the mathematical rigor [9]. It is shown in Chapter 9.5 of the cited book that the acceleration of a chosen material point of the ISA can be decomposed into a component that is parallel with the ISA due to the translatory part of the raccording motion and another component that is related to the rolling about the ISA. It is also stated that the latter part is perpendicular to the common tangent plane of the moving and fixed axodes. However, the proof of this statement refers to the solution of a planar rolling problem, when the body-fixed contact point passes through a cusp of its trajectory (cf. Fig. 8), implying that its acceleration is perpendicular to the tangent plane. This is certainly true, but the exact generalization to spatial motions is not given explicitly in [9].

The goal of the present paper is to find a straightforward, time-based derivation that establishes the relation between the instantaneous acceleration of points along the ISA and the apparent motion of the ISA on the fixed axode. The novelty of the proposed approach lies in the fact that the results are derived using Euler’s rigid body formulas (1), (2) and that the concept of pole changing velocity is generalized to spatial motions.

### Organization of the paper

The paper is organized as follows: Sect. 2 deals with the definition and extension of the notion of pole to spatial motions. In Sect. 3, the formula of pole changing velocity is derived and the obtained result is interpreted. Further connections are established between the pole changing velocity $\mathbf {u}$ and the pole acceleration $\mathbf {a}_P$ in Sect. 4. It is shown in Sect. 5 as a corollary that the finite-time (continuous) motions of rigid bodies can be classified into three categories: planar motion, spherical motion and general raccording motion. Although this result (attributed to Painlevé in [9]) is well known in kinematics, the present paper provides a proof that is different from the conventional geometric approach. Section 6 illustrates the derived results via numerical examples, and the conclusions are drawn in Sect. 7.

## Formulation of the geometric pole’s position

**Fig. 5:**
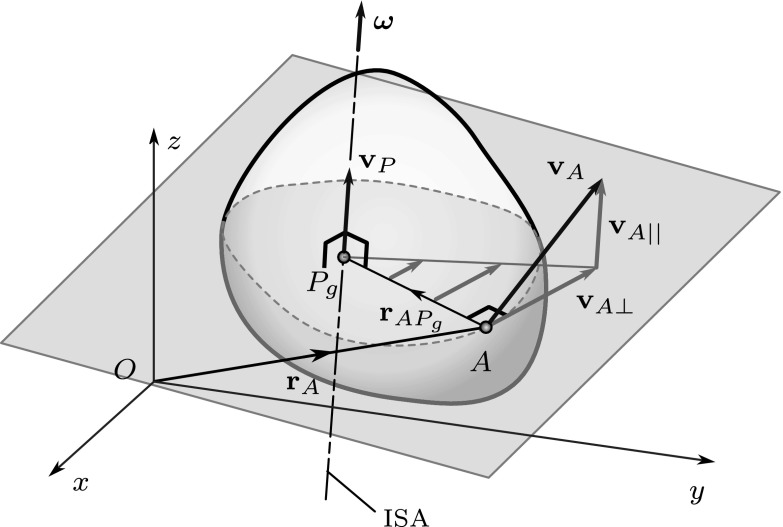
Determination of the point $P_g$ on the instantaneous screw axis. The velocity vector $\mathbf {v}_P$ of the material point *P* coinciding with $P_g$ is shown

If a rigid body has nonzero angular velocity $\varvec{\omega }$, there exists a so-called instantaneous screw axis (ISA) such that the ISA is parallel with $\varvec{\omega }$ and the material points coinciding with the ISA have no velocity component perpendicular to $\varvec{\omega }$. Although these material points may have a velocity component parallel to $\varvec{\omega }$, they will also be referred to as poles for the sake of simplicity. Just as in the planar case, we distinguish between the pole *P* (material point) and the geometric pole $P_g$ (whose position is defined by geometric and additional kinematic conditions). If the velocity $\mathbf {v}_A$ of a reference point *A* of the body is known, one can determine the geometric position of a point $P_g$ on the ISA. $P_g$ is searched for on the plane that is perpendicular to $\varvec{\omega }$ and passes through point *A*, as shown in Fig. 5. The coinciding material point is denoted by *P*.

According to [5, 6, 9], the location of point $P_g$ can be determined as follows. Using Euler’s formula,

4 $$\begin{aligned} \mathbf {v}_{P} = \mathbf {v}_A + \varvec{\omega }\times \mathbf {r}_{AP}. \end{aligned}$$

This formula establishes the relation between the velocities of two *material* points of the body. To obtain the location of points on the ISA, the previous formula is premultiplied by $\varvec{\omega }$:

5 $$\begin{aligned} \varvec{\omega }\times \mathbf {v}_P = \varvec{\omega }\times \mathbf {v}_A + \varvec{\omega }\times (\varvec{\omega }\times \mathbf {r}_{AP}). \end{aligned}$$

The expansion of the vector triple product leads to

6 $$\begin{aligned} \varvec{\omega }\times \mathbf {v}_P = \varvec{\omega }\times \mathbf {v}_A + \varvec{\omega }(\varvec{\omega }\cdot \mathbf {r}_{AP}) - \mathbf {r}_{AP} \omega ^2, \end{aligned}$$

where the dot denotes scalar product.

Point *A* is a chosen *reference point* of the rigid body—a material point, while point *P* is unknown yet. In a certain instant *t*, we want to find the locus of a point $P_g$ such that the material point *P* coinciding with it fulfills the following two conditions: $\varvec{\omega }(t) \parallel \mathbf {v}_P(t)$ and $\mathbf {r}_{AP}(t) \perp \varvec{\omega }(t)$, i.e.,

7 $$\begin{aligned} \mathbf {0} = \varvec{\omega }(t)\times \mathbf {v}_A(t) - \mathbf {r}_{AP_g}(t)\ \omega ^2(t) \quad \Rightarrow \quad \mathbf {r}_{AP_g}(t) = \frac{\varvec{\omega }(t)\times \mathbf {v}_A(t)}{\omega ^2(t)}. \end{aligned}$$

Since the position of point $P_g$ depends on additional conditions besides Equation (4), the resulting vector–scalar function $\mathbf {r}_{AP_g}(t)$ describes the motion of the *geometric pole*. Thus, while the geometric pole and the pole coincide in the considered instant *t*: $\mathbf {r}_{AP}(t) = \mathbf {r}_{AP_g}(t)$, their derivatives are different in general: ${\dot{\mathbf {r}}}_{AP}(t) \ne {\dot{\mathbf {r}}}_{AP_g}(t)$.

If $\mathbf {v}_A \perp \varvec{\omega }$, the velocity of the material points along the ISA is zero; thus, the rigid body undergoes instantaneous rotation. In this case, the ISA is referred to as instantaneous axis of rotation (IAR). Equation (7) is valid in the planar case, too, when the points of the rigid body move in parallel planes that are perpendicular to the angular velocity $\varvec{\omega }$. The same velocities and accelerations can be seen in these planes. Thus, the motion can be represented in a single, properly chosen plane, as shown in Fig. 3.

**Fig. 6:**
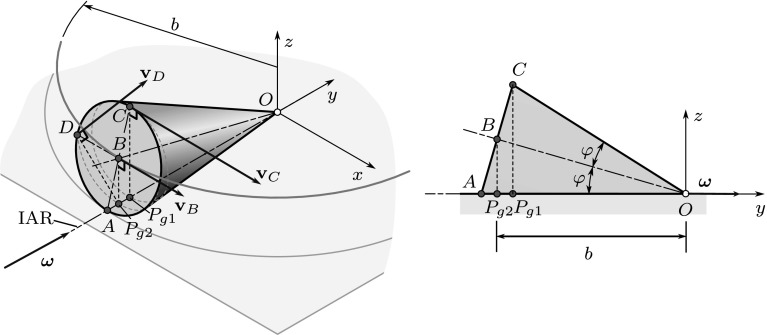
Different geometric poles can be assigned to different reference points. The geometric poles corresponding to points *C* and *D* are $P_{g1}$ and $P_{g2}$, respectively, while point *A* just coincides with a point on the instantaneous axis of rotation

It is important to mention that in general different geometric poles are assigned to different reference points of the rigid body. This is due to the fact that point $P_g$ is searched for in the plane that is perpendicular to $\varvec{\omega }$ and passes through the reference point, according to (7). As a consequence, the position of the geometric pole may vary along the ISA (or IAR) during the motion of the body, depending on the choice of the reference point. Figure 6 shows an example that illustrates the rolling of a cone, i.e., a spherical motion.

In principle, if the geometric pole is chosen according to (7), it moves in space during the motion of the body, forming a fixed polode (space curve). In the instant shown in Fig. 6, the reference point *A* just coincides with the geometric pole assigned to it, but at later instants—as the projection of *A* gets closer to the center point *O* (see points *D* and *C* and their projections)—the geometric pole also moves closer to *O* along the IAR. The points of the moving polode could be defined via the material points that coincide with the points of the fixed polode.

Note, however, that the polode curves defined this way depend on the choice of the reference point. If a point on the perimeter of the cone’s base (*A*, *C* or *D*) is chosen as a reference point, the fixed polode curves defined by (7) meander on the $z = 0$ plane. The distance of the points of these polode curves from the center point *O* varies during the motion between $b - \overline{P_{g1}P_{g2}}$ and $b + \overline{P_{g1}P_{g2}}$, with different phases for the different reference points.

There is a more practical procedure in the case of spherical motions: In this case, it is possible to assign the geometric pole to a reference point in such a way that the distance of these points from the fixed center point *O* is the same. In Fig. 6, the points *A*, *C* and *D* are at the same distance from *O*, so the same geometric pole (just coinciding with *A* in the figure) can be assigned to all the points on the perimeter of the base of the cone. As a consequence, both the fixed and moving polodes will be circles in this special case.

Similarly, if point *B* is chosen to be the reference point, the distance of its projection ($P_{g2}$) from *O* does not change, so one obtains circular polodes, again.

We can conclude that there are several possibilities for the assignment of the geometric pole to the reference point; thus, the generalization of the polode curves to the spatial case is usually impractical. This is why the present paper focuses mainly on the instantaneous properties of the motion instead of the geometric objects corresponding to finite-time motion.

## Generalization of pole changing velocity

### Formal derivation of pole changing velocity

**Fig. 7:**
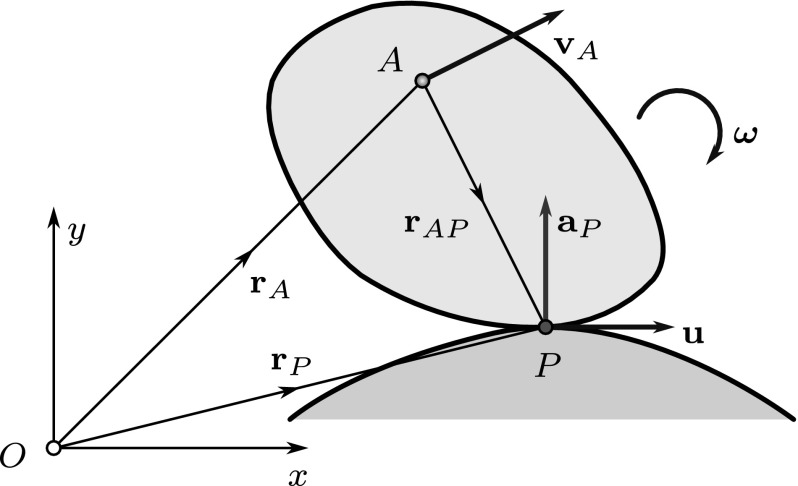
Pole changing velocity and pole acceleration in the planar case

Let us assume that the angular velocity $\varvec{\omega }$ of the rigid body, the angular acceleration $\varvec{\alpha }$ of the rigid body and the velocity and acceleration of point *A* are known. The position of the geometric pole $P_g$ can be given relative to point *A* by Eq. (7); consequently, $\mathbf {r}_{P_g}(t) = \mathbf {r}_A(t) + \mathbf {r}_{AP_g}(t)$ (Figs. 5, 7).

The pole changing velocity is—by definition—the derivative of $\mathbf {r}_{P_g}(t)$ with respect to time:

$$\begin{aligned} \mathbf {u}\equiv {\dot{\mathbf {r}}}_{P_g}(t) = {\dot{\mathbf {r}}}_A(t) +{\dot{\mathbf {r}}}_{AP_g}(t), \end{aligned}$$

where $\mathbf {r}_{AP_g} = (\varvec{\omega }\times \mathbf {v}_A)/\omega ^2$, according to (7). Since this vector is related to the position of the geometric pole, neither its magnitude nor its direction is constant in the general case.

Vector $\mathbf {u}$ characterizes the apparent motion of the ISA; thus, it must lie in the common tangent plane of the moving and fixed axodes at $P_g$. In the general case, the direction of the tangent plane varies along the ISA (Fig. 4).

Exploiting the differentiation rule of fractions, one obtains

$$\begin{aligned} \mathbf {u}= \mathbf {v}_A + \frac{(\varvec{\alpha }\times \mathbf {v}_A)}{\omega ^2}+\frac{(\varvec{\omega }\times \mathbf {a}_A)}{\omega ^2}-2 (\varvec{\omega }\cdot \varvec{\alpha }) \frac{(\varvec{\omega }\times \mathbf {v}_A) }{\omega ^4}, \end{aligned}$$

where $\varvec{\alpha }= {\dot{\varvec{\omega }}}$ is the angular acceleration, while $\mathbf {a}_A = {\dot{\mathbf {v}}}_A$ is the acceleration of point *A*.

The velocity and acceleration of point *A* can be expressed as $\mathbf {v}_A = \mathbf {v}_P + \varvec{\omega }\times \mathbf {r}_{PA}$ and $\mathbf {a}_A = \mathbf {a}_P + \varvec{\alpha }\times \mathbf {r}_{PA} +\varvec{\omega }\times (\varvec{\omega }\times \mathbf {r}_{PA})$, respectively. Note that $\mathbf {r}_{PA}$ denotes the position vector of the material pole, so $|\mathbf {r}_{PA}(t)|$ is constant and Euler’s formulas are valid in this case. Vector $\mathbf {a}_P$ denotes the acceleration of the material point *P* that coincides the geometric pole $P_g$, i.e., this is the *pole acceleration*.

After substitution, one obtains

8 $$\begin{aligned} \mathbf {u}&= \mathbf {v}_P + \varvec{\omega }\times \mathbf {r}_{PA} + \frac{\varvec{\alpha }\times (\varvec{\omega }\times \mathbf {r}_{PA})}{\omega ^2} +\frac{\varvec{\omega }\times \mathbf {a}_P}{\omega ^2}+\frac{\varvec{\omega }\times (\varvec{\alpha }\times \mathbf {r}_{PA})}{\omega ^2}\nonumber \\ {}&\quad +\frac{\varvec{\omega }\times (\varvec{\omega }\times (\varvec{\omega }\times \mathbf {r}_{PA}))}{\omega ^2}-2 (\varvec{\omega }\cdot \varvec{\alpha }) \frac{\varvec{\omega }\times (\varvec{\omega }\times \mathbf {r}_{PA})}{\omega ^4}. \end{aligned}$$

$\mathbf {v}_P$ is parallel to $\varvec{\omega }$, by the definition of point *P*. Moreover, it is known that $\mathbf {r}_{PA} \perp \varvec{\omega }$, i.e., $\varvec{\omega }\cdot \mathbf {r}_{PA} =0$ since *P* is in the plane that passes through point *A* and is perpendicular to $\varvec{\omega }$. This property can be used for the simplification of the formula of $\mathbf {u}$. Applying the vector triple product expansion, we obtain

9 $$\begin{aligned} \begin{aligned} \frac{\varvec{\alpha }\times (\varvec{\omega }\times \mathbf {r}_{PA})}{\omega ^2}&= \frac{\varvec{\omega }(\varvec{\alpha }\cdot \mathbf {r}_{PA})}{\omega ^2}-\frac{\mathbf {r}_{PA}(\varvec{\alpha }\cdot \varvec{\omega })}{\omega ^2},\\ \frac{\varvec{\omega }\times (\varvec{\alpha }\times \mathbf {r}_{PA})}{\omega ^2}&= \frac{\varvec{\alpha }(\varvec{\omega }\cdot \mathbf {r}_{PA})}{\omega ^2}-\frac{\mathbf {r}_{PA}(\varvec{\alpha }\cdot \varvec{\omega })}{\omega ^2}=-\frac{\mathbf {r}_{PA}(\varvec{\alpha }\cdot \varvec{\omega })}{\omega ^2},\\ \frac{\varvec{\omega }\times (\varvec{\omega }\times (\varvec{\omega }\times \mathbf {r}_{PA}))}{\omega ^2}&= \frac{\varvec{\omega }}{\omega ^2} \times \bigg (\varvec{\omega }(\varvec{\omega }\cdot \mathbf {r}_{PA})-\mathbf {r}_{PA}\omega ^2\bigg ) = -\varvec{\omega }\times \mathbf {r}_{PA},\\ -2 (\varvec{\omega }\cdot \varvec{\alpha }) \frac{\varvec{\omega }\times (\varvec{\omega }\times \mathbf {r}_{PA})}{\omega ^4}&= -2 (\varvec{\omega }\cdot \varvec{\alpha }) \frac{\varvec{\omega }(\varvec{\omega }\cdot \mathbf {r}_{PA})-\mathbf {r}_{PA}\omega ^2}{\omega ^4}=2\frac{\mathbf {r}_{PA} (\varvec{\alpha }\cdot \varvec{\omega })}{\omega ^2}. \end{aligned} \end{aligned}$$

After substitution into (8), most of the terms vanish, except for three—two of them are parallel with the angular velocity, while the third one is perpendicular to it:

10 $$\begin{aligned} \mathbf {u}= \mathbf {v}_P + \frac{\varvec{\omega }(\varvec{\alpha }\cdot \mathbf {r}_{PA})}{\omega ^2}+ \frac{\varvec{\omega }\times \mathbf {a}_P}{\omega ^2}. \end{aligned}$$

This formula is valid for arbitrary spatial motions.

### Interpretation of the obtained result

Equation (10) shows that the pole changing velocity generally depends on the chosen reference point *A*, in accordance with Sect. 2 and Fig. 6.

In the case of *planar motion*, $\mathbf {v}_P = \mathbf {0}$ and $\varvec{\alpha }\perp \mathbf {r}_{PA}$; thus, the first two terms of (10) vanish. Consequently,

11 $$\begin{aligned} \mathbf {u}= \frac{\varvec{\omega }\times \mathbf {a}_P}{\omega ^2} \quad \Rightarrow \quad \mathbf {u}\perp \varvec{\omega }\quad \text{ and } \quad \mathbf {u}\perp \mathbf {a}_P, \end{aligned}$$

independently on the reference point, as it is well known from the literature [9]. This case is shown in Fig. 7.

In the case of *spatial motions*, the term $\varvec{\omega }(\varvec{\alpha }\cdot \mathbf {r}_{PA})/\omega ^2$ is nonzero only if the angular acceleration $\varvec{\alpha }$ has a component parallel to $\mathbf {r}_{PA}$ and—consequently—perpendicular to $\varvec{\omega }$. Since this term originates from Eq. (9), it means that the velocity of the reference point ($\mathbf {v}_A = \varvec{\omega }\times \mathbf {r}_{PA}$) must have a component perpendicular to $\varvec{\alpha }$ in this case.

For the further analysis, recall that even in the most general case of raccording motion, the geometric positions of the ISA define the moving and fixed axodes. These are ruled surfaces with a common tangent plane [9]. As a consequence, the angular velocity $\varvec{\omega }$ and the pole changing velocity $\mathbf {u}$ are always parallel to this tangent plane.

In the case of *spherical motion*, one of the points of the ISA has zero velocity and acceleration. Thus, the apparent motion of the ISA can be characterized by the change of its direction. Since the angular velocity $\varvec{\omega }$ is parallel to the ISA, the change of direction of $\varvec{\omega }$—described by the component of $\varvec{\alpha }$ that is perpendicular to $\varvec{\omega }$—must take place parallel to the tangent plane. Thus, $\varvec{\omega }(\varvec{\alpha }\cdot \mathbf {r}_{PA})/{\omega ^2}$ is nonzero only if $\mathbf {r}_{PA}$ has a component parallel with the tangent plane. In this case, the velocity of the reference point ($\mathbf {v}_A = \varvec{\omega }\times \mathbf {r}_{PA}$) has a component perpendicular to the tangent plane.

To visualize this result, see Fig. 6, where the $z=0$ plane is the tangent plane. If one chooses a reference point on the perimeter of the base of the cone (e.g., *A*, *D* or *C*), the radial position (distance from *O*) of the corresponding geometric pole will vary during the rolling of the cone. Clearly, the extremal positions of the geometric pole correspond to the configurations when the reference point is just on the tangent plane (point *A*) or at the upper position (point *C*). The pole changing velocities of the corresponding poles ($P_{g1}$ and the point coinciding *A*) have no component parallel with $\varvec{\omega }$, in accordance with $\mathbf {r}_{AA} = \mathbf {0}$ and that $\mathbf {r}_{P_{g1}A}$ is perpendicular to the contact plane. However, if point *D* is the reference point, the corresponding geometric pole ($P_{g2}$) is transferred closer to the center point *O* during the motion, i.e., its pole changing velocity has a component parallel to $\varvec{\omega }$.

A well-defined generalization of pole changing velocity should not depend on the position of the arbitrarily chosen reference point *A*. Thus, for the examination of the instantaneous acceleration of a pole point *P*, the following procedure is proposed: After choosing an arbitrary reference point, the corresponding geometric pole $P_g$ on the ISA can be found using (7). Once the position of the ISA is known, one can select a material point *P* of interest on it, based on practical considerations. Then, formally, the point *P* itself can be used as the reference point. Using this procedure, the pole can be uniquely identified and its acceleration—together with the pole changing velocity of the coinciding geometric pole—can be determined. With the choice $A \equiv P$ in (10), one arrives at $\mathbf {r}_{PP} = \mathbf {0}$. Thus, the reference point-independent formula of the pole changing velocity is

12 $$\begin{aligned} \mathbf {u}= \mathbf {v}_P + \frac{\varvec{\omega }\times \mathbf {a}_P}{\omega ^2}. \end{aligned}$$

**Fig. 8:**
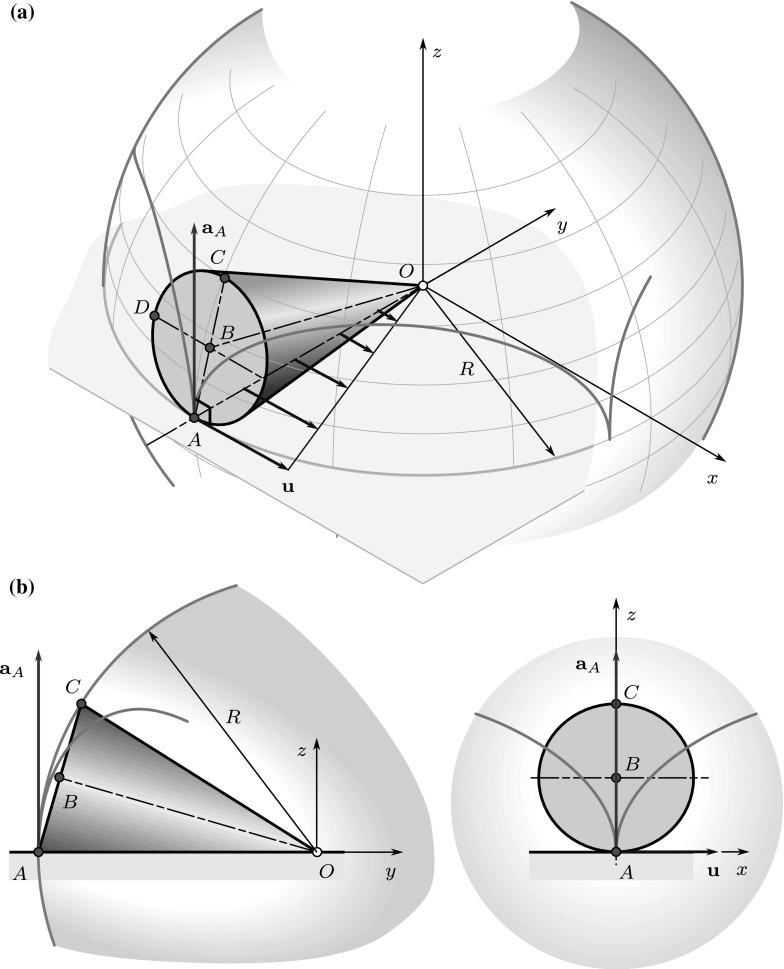
Example of spherical motion: rolling of a cone on the (*xy*) plane. The IAR is found along the *y*-axis. Each point of the IAR has different pole changing velocity

Consequently,

13 $$\begin{aligned} \mathbf {u}= \frac{\varvec{\omega }\times \mathbf {a}_P}{\omega ^2} \end{aligned}$$

is valid for spatial *rotational motions* ($\mathbf {v}_P = \mathbf {0}$), too, just as in the planar case. Thus, during rotation the pole changing velocity is perpendicular to the angular velocity and the acceleration of point *P*, as it is illustrated in Fig. 8.

## Pole acceleration

The importance of the notion of pole changing velocity lies in the fact that it is related to the acceleration of the pole $\mathbf {a}_P$. However, Eq. (12) does not provide any information about the mutual direction of the angular velocity $\varvec{\omega }$ and the acceleration $\mathbf {a}_P$.

In the present section, we show that in the case of planar motion or spherical motion, the relation

14 $$\begin{aligned} \mathbf {a}_P = \mathbf {u}\times \varvec{\omega }\end{aligned}$$

is fulfilled.

Taking the cross product of both sides of (10) by $\varvec{\omega }$, the first two terms cancel out, independently on the choice of the reference point since $\mathbf {v}_P \parallel \varvec{\omega }$. Thus,

$$\begin{aligned} \varvec{\omega }\times \mathbf {u}= \frac{\varvec{\omega }\times (\varvec{\omega }\times \mathbf {a}_P)}{\omega ^2}. \end{aligned}$$

The right-hand side can be rewritten using the vector triple product expansion:

$$\begin{aligned} \varvec{\omega }\times \mathbf {u}= \frac{\varvec{\omega }}{\omega }\left( \frac{\varvec{\omega }}{\omega }\cdot \mathbf {a}_P\right) -\mathbf {a}_P. \end{aligned}$$

Since the first term on the right-hand side is that component of $\mathbf {a}_P$ which is parallel with $\varvec{\omega }$ (denoted by $\mathbf {a}_{P||}$),

15 $$\begin{aligned} \mathbf {u}\times \varvec{\omega }= \mathbf {a}_P - \mathbf {a}_{P||}. \end{aligned}$$

This result is valid for arbitrary spatial motions.

In the case of *planar motion*, all the accelerations are perpendicular to the angular velocity, i.e., $\mathbf {a}_P = \mathbf {u}\times \varvec{\omega }$ is fulfilled.

To extend the derivation to *spherical motions*, let us compare the accelerations of two points $P_1$ and $P_2$ along the IAR:

16 $$\begin{aligned} \mathbf {a}_{P2}-\mathbf {a}_{P1} = \varvec{\alpha }\times \mathbf {r}_{P1P2} + \varvec{\omega }\times (\varvec{\omega }\times \mathbf {r}_{P1P2}), \end{aligned}$$

where $\mathbf {r}_{P1P2} \parallel \varvec{\omega }$; thus, the first term on the right-hand side is perpendicular to both the angular velocity and the angular acceleration, while the second term is $\mathbf {0}$.

Consequently, $\mathbf {a}_{P2}$ and $\mathbf {a}_{P1}$ can differ only in a component that is perpendicular to $\varvec{\omega }$. Since in the case of *spherical motions* the IAR has a point with zero acceleration, $\mathbf {a}_{P||} = \mathbf {0}$ is fulfilled and

17 $$\begin{aligned} \mathbf {a}_P = \mathbf {u}\times \varvec{\omega }. \end{aligned}$$

Let us remark that similar results were derived in [9], using a different way of thoughts, without the generalization of the pole changing velocity.

## Finite-time rotational motions

A rigid body exhibits a finite-time rotational motion when the moving axode rolls on the fixed axode without slip, i.e., $\mathbf {v}_P = \mathbf {0}$ and $\mathbf {a}_{P||} = \mathbf {0}$. It follows from the previous results that in this case

18 $$\begin{aligned} \mathbf {a}_P = \mathbf {u}\times \varvec{\omega }. \end{aligned}$$

According to the previous sections, $\mathbf {u}$ must be in the tangent plane, and it is perpendicular to the IAR in the case of rotational motion, i.e., if $\mathbf {v}_P = \mathbf {0}$ [see (12)]. Thus, in the case of rotational motions, $\mathbf {a}_P$ is perpendicular to the tangent plane. As is shown in Eq. (16), the accelerations of two points along the IAR $\mathbf {a}_{P2}$ and $\mathbf {a}_{P1}$ can differ only in a component

19 $$\begin{aligned} \mathbf {a}_{P2} - \mathbf {a}_{P1} = \varvec{\alpha }\times \mathbf {r}_{P1P2}, \end{aligned}$$

where $\mathbf {r}_{P1P2} \parallel \varvec{\omega }$. Consequently, the following cases can be distinguished:

- If $\varvec{\alpha }\parallel \varvec{\omega }$, the acceleration of points and the pole changing velocity are constant along the IAR. This motion corresponds to the rolling of a cylinder, i.e., to planar motion.
- If $\mathbf {a}_{P1} \parallel \varvec{\alpha }\times \varvec{\omega }$, there must be a point $P_2$ along the IAR with zero acceleration. This case corresponds to the rolling of a cone, i.e., to spherical motion (Fig. 8). Recall that if the acceleration is zero at a point of the IAR, the corresponding pole changing velocity [cf. (13)] is also zero. Thus, we turn to the analysis of the pole changing velocity, again. By expressing the pole changing velocities corresponding to the points $P_1$ and $P_2$ in (19) by (12), one obtains^1^
  20 $$\begin{aligned} \mathbf {u}_2 = \mathbf {u}_1 + \frac{\varvec{\omega }\times (\varvec{\alpha }\times \mathbf {r}_{P1P2})}{\omega ^2}. \end{aligned}$$
  If the same tangent plane is spanned by $\mathbf {u}$ and $\varvec{\omega }$ at all points of the IAR, the direction of $\mathbf {u}$ is also the same along the IAR. According to (20), the magnitude of pole changing velocity varies linearly along the IAR. Thus, the geometric point with zero pole changing velocity (coinciding the material point with zero acceleration) can be found in a straightforward way, as is shown in Fig. 8. However, there are more general cases, too, when different tangent planes can be found for different points along the IAR, as illustrated in Fig. 4. For the further analysis, we expand the vector triple product in (20):
  21 $$\begin{aligned} \mathbf {u}_2 = \mathbf {u}_1 + \varvec{\alpha }\frac{\varvec{\omega }\cdot \mathbf {r}_{P1P2}}{\omega ^2} - \mathbf {r}_{P1P2} \frac{\varvec{\alpha }\cdot \varvec{\omega }}{\omega ^2}. \end{aligned}$$
  We search for the point $P_2$ to which zero pole changing velocity $\mathbf {u}_2$ is assigned. Multiplying the previous formula by $\varvec{\omega }$,
  22 $$\begin{aligned} \varvec{\omega }\times \mathbf {u}_2 = \varvec{\omega }\times \mathbf {u}_1 + (\varvec{\omega }\times \varvec{\alpha }) \frac{\varvec{\omega }\cdot \mathbf {r}_{P1P2}}{\omega ^2} - (\varvec{\omega }\times \mathbf {r}_{P1P2}) \frac{\varvec{\alpha }\cdot \varvec{\omega }}{\omega ^2}. \end{aligned}$$
  The last term is zero since $\varvec{\omega }\parallel \mathbf {r}_{P1P2}$. According to the condition $\mathbf {u}_2 = \mathbf {0}$, the left-hand side of the equation is also zero. Consequently,
  23 $$\begin{aligned} -\varvec{\omega }\times \mathbf {u}_1 = (\varvec{\omega }\times \varvec{\alpha }) \frac{\varvec{\omega }\cdot \mathbf {r}_{P1P2}}{\omega ^2}. \end{aligned}$$
  It means that to find the vector $\mathbf {r}_{P1P2}$, the condition $\varvec{\omega }\times \mathbf {u}_1 \parallel \varvec{\omega }\times \varvec{\alpha }$ must be fulfilled. Since $\mathbf {u}$ and $\varvec{\omega }$ span the tangent plane of the axodes, it means that the angular acceleration $\varvec{\alpha }$ must be also in the tangent plane at a point along the IAR during spherical motions.
- The aforementioned condition is not always fulfilled. In the case of the more general raccording motion (Fig. 4), the direction of the tangent plane’s normal vector varies along the IAR. Consequently, the direction of the pole changing velocity and the acceleration of material points also varies along the IAR. If a pole point has an acceleration component that is perpendicular to $\varvec{\alpha }\times \varvec{\omega }$, there is no point along the ISA that has zero acceleration. There exists a point *P* that has no acceleration component parallel with $\varvec{\alpha }\times \varvec{\omega }$. This point is referred to as striction point [9].

## Numerical examples

### Rolling of a cone

**Fig. 9:**
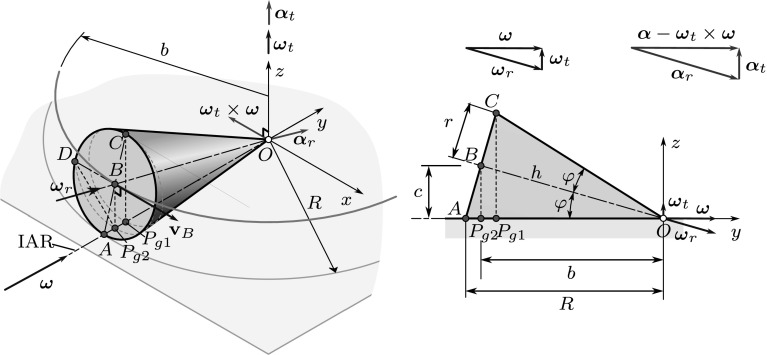
Illustration of the numerical example: rolling of a cone on a horizontal surface

Consider the rolling cone depicted in Fig. 9.

Let the radius of the base, the height and the slant height of the cone be $r = 0.14\,\mathrm{m}$, $h = 0.48\,\mathrm{m}$ and $R = 0.5\,\mathrm{m}$, respectively. The apex of the cone is fixed in the origin *O*. The cone rolls on the *xy* plane without slipping. The *x* components of the velocity and acceleration of point *B* are $v_{Bx} = 1.92\,\mathrm{m/s}$ and $a_{Bx} = 1\,\mathrm{m/s}^2$.

The angle $\varphi$ can be determined as

24 $$\begin{aligned} \sin (\varphi ) = \frac{r}{R} = 0.28,\quad \cos (\varphi ) = \frac{h}{R} = 0.96. \end{aligned}$$

The position vector of point *B* is

25 $$\begin{aligned} {\mathbf {r}}_{OB} = \begin{bmatrix} 0\\ -h \cos (\varphi )\\ h\sin (\varphi ) \end{bmatrix} \equiv \begin{bmatrix} 0\\ -b\\ c \end{bmatrix}. \end{aligned}$$

The moving axode just coincides with the surface of the cone, while the fixed axode is the *xy* plane. The instantaneous axis of rotation and the angular velocity are parallel with the *y*-axis and the tangent plane of the axodes is the *xy* plane, itself. Exploiting that $\mathbf {v}_B = \varvec{\omega }\times \mathbf {r}_{OB}$, one obtains

26 $$\begin{aligned} \varvec{\omega }= \begin{bmatrix} 0\\ \omega _{y}\\ 0 \end{bmatrix} = \begin{bmatrix} 0\\ 14.2857 \\ 0 \end{bmatrix} {\frac{{\hbox {rad}}}{{\hbox {s}}}}. \end{aligned}$$

This angular velocity vector can be decomposed into two components: the angular velocity of transportation $\varvec{\omega }_t$ and the relative angular velocity $\varvec{\omega }_r$: $\varvec{\omega }= \varvec{\omega }_t + \varvec{\omega }_{r}$ [26]. The relative angular velocity describes the rotation of the cone about its symmetry axis *OB*, while the angular velocity of transportation characterizes the rotation of the symmetry axis about the *z*-axis. Using the given data,

27 $$\begin{aligned} \varvec{\omega }_t = \begin{bmatrix} 0\\0\\ \frac{v_{Bx}}{b} \end{bmatrix} = \begin{bmatrix} 0\\0\\ 4.1\dot{6} \end{bmatrix}\, {{\frac{{\hbox {rad}}}{{\hbox {s}}}}}\quad \text{ and } \quad \varvec{\omega }_{r} = \begin{bmatrix} 0\\14.2857\\4.1\dot{6} \end{bmatrix}\, {\frac{\mathrm{rad}}{\mathrm{s}}}. \end{aligned}$$

Note that due to the constraint of rolling, the ratio of the magnitudes of these vectors is $|\varvec{\omega }_t|/|\varvec{\omega }_r| = \sin (\varphi ) = r/R$.

The velocities of points *C* and *D* can be determined by Euler’s formula, exploiting that the velocity of the points along the *y*-axis is zero:

28 $$\begin{aligned} \mathbf {v}_C = \mathbf {v}_A + \varvec{\omega }_{} \times \mathbf {r}_{AC} = \varvec{\omega }_{} \times \mathbf {r}_{AC}= \begin{bmatrix} 2r\cos (\varphi ) \omega _{y}\\0\\0 \end{bmatrix}= 2 \mathbf {v}_B =\begin{bmatrix} 3.84\\0\\0 \end{bmatrix}\, {\frac{\mathrm{m}}{\mathrm{s}}}, \end{aligned}$$

29 $$\begin{aligned} \mathbf {v}_D = \varvec{\omega }_{} \times \mathbf {r}_{AD} = \begin{bmatrix} 0\\\omega _{y}\\0 \end{bmatrix} \times \begin{bmatrix} -r\\ \mathbf {r}\sin (\varphi )\\ \mathbf {r}\cos (\varphi ) \end{bmatrix} = \begin{bmatrix} r\cos (\varphi ) \omega _{y}\\0\\ \mathbf {r}\omega _{y} \end{bmatrix} = \begin{bmatrix} 1.92\\0\\2 \end{bmatrix}\, {\frac{\mathrm{m}}{\mathrm{s}}}. \end{aligned}$$

Thus, the positions of the poles assigned to these points are

30 $$\begin{aligned} \mathbf {r}_{CPg1} = \frac{\varvec{\omega }_{}\times \mathbf {v}_C}{\omega _2^2} = \begin{bmatrix} 0\\ 0\\ -2r\cos (\varphi ) \end{bmatrix} = \begin{bmatrix} 0\\ 0\\ -0.2688 \end{bmatrix}\,\mathrm{m}, \end{aligned}$$

and

31 $$\begin{aligned} \mathbf {r}_{DPg2} = \frac{\varvec{\omega }_{}\times \mathbf {v}_D}{\omega _2^2} = \begin{bmatrix} r\\ 0\\ -r\cos (\varphi ) \end{bmatrix} = \begin{bmatrix} 0.14\\ 0\\ -0.1344 \end{bmatrix}\,\mathrm{m}. \end{aligned}$$

Moreover, point *A* coincides with a geometric pole that will be denoted by $A_g$ as follows. Points $A_g$, $P_{g1}$ and $P_{g2}$ apparently move in circular paths with angular velocity $\varvec{\omega }_t$. The radii of these circles are $R_{A_g} = R = 0.5\,\mathrm{m}$, $R_{P_{g1}} = 0.4216\,\mathrm{m}$, and $R_{P_{g2}} = b = h \cos (\varphi ) = 0.4216\,\mathrm{m}$, respectively. The corresponding pole changing velocities are all parallel with the *x*-axis, pointing in the positive direction. Their magnitudes can be determined by multiplying the radii of the circles by the magnitude of $\varvec{\omega }_t$: $|\varvec{\omega }_t| = 4.1\dot{6}\,{{{\mathrm{rad}}/{\mathrm{s}}}}$. Thus,

32 $$\begin{aligned} \mathbf {u}_{A_g} = \begin{bmatrix} 2.08\dot{3}\\0\\0 \end{bmatrix} \, {\frac{\mathrm{m}}{\mathrm{s}}},\quad \mathbf {u}_{P_{1g}} = \begin{bmatrix} 1.75\dot{6}\\0\\0 \end{bmatrix} \, {\frac{\mathrm{m}}{\mathrm{s}}},\quad \mathbf {u}_{P_{2g}} = \begin{bmatrix} 1.92\\0\\0 \end{bmatrix} \, {\frac{\mathrm{m}}{\mathrm{s}}}. \end{aligned}$$

Now, exploiting (17), the accelerations of the corresponding material points *A*, $P_1$ and $P_2$ can be determined: $\mathbf {a}_{A} = \mathbf {u}_{A_g} \times \varvec{\omega }$, $\mathbf {a}_{P_{1}} = \mathbf {u}_{P_{g1}} \times \varvec{\omega }$ and $\mathbf {a}_{P_{2}} = \mathbf {u}_{P_{g2}} \times \varvec{\omega }$; thus,

33 $$\begin{aligned} \mathbf {a}_{A} = \begin{bmatrix} 0\\0\\29.7619 \end{bmatrix} \, {\frac{\mathrm{m}}{\mathrm{s}^2}},\quad \mathbf {a}_{P_{1}} = \begin{bmatrix} 0\\0\\25.0952 \end{bmatrix} \, {\frac{\mathrm{m}}{\mathrm{s}^2}},\quad \mathbf {a}_{P_{2}} = \begin{bmatrix} 0\\0\\27.4286 \end{bmatrix} \, {\frac{\mathrm{m}}{\mathrm{s}^2}}. \end{aligned}$$

To check these results, the accelerations will be determined by the direct use of Euler’s formulas, too. It is known that the acceleration of point *O* is zero: $\mathbf {a}_O = \mathbf {0}$, and the components of the angular acceleration are unknown: $\varvec{\alpha }= [\alpha _{x}\, \alpha _{y}\,\alpha _{z}]^T$. Since point *B* moves in a circular path of radius *b*,

34 $$\begin{aligned} \mathbf {a}_B = \begin{bmatrix} a_{Bx}\\ \frac{v_B^2}{b}\\0 \end{bmatrix}= \begin{bmatrix} 1\\8\\0 \end{bmatrix} \, {\frac{\mathrm{m}}{\mathrm{s}^2}}. \end{aligned}$$

$\mathbf {a}_B$ can be expressed by Euler’s formula, too:

35 $$\begin{aligned} \mathbf {a}_B = \mathbf {a}_O + \varvec{\alpha }_{}\times \mathbf {r}_{OB} + \varvec{\omega }_{}\times (\varvec{\omega }_{}\times \mathbf {r}_{OB}) = \begin{bmatrix} c \alpha _{y} + b \alpha _{z}\\ -c \alpha _{x}\\ -b \alpha _{x} - c\omega _{y}^2 \end{bmatrix}. \end{aligned}$$

Comparing the two expressions of $\mathbf {a}_B$, one obtains that $\alpha _{x}$ can be calculated using either the *y* or the *z* component of the equation, since $\omega _{y}$ and $v_B$ are related by the constraint of rolling:

36 $$\begin{aligned} \alpha _{x}&= -\frac{v_B^2}{cb} = -59.5238~{\frac{\mathrm{rad}}{\mathrm{s}^2}},\quad \text{ and }\nonumber \\ \alpha _{x}&= -\frac{c\omega _{y}^2}{b}= -59.5238\ {\frac{\mathrm{rad}}{\mathrm{s}^2}}. \end{aligned}$$

To determine $\alpha _{y}$ and $\alpha _{z}$, we have only a single equation left: $c \alpha _{y} + b \alpha _{z} = a_{Bx}$. So, an additional equation is necessary. We can exploit that—as it was derived in Sect. 5—in the case of spherical motions $\varvec{\alpha }$ must be parallel with the tangent plane of the axodes, i.e., $\alpha _z = 0$. Consequently,

37 $$\begin{aligned} \alpha _{y}= \frac{a_{Bx}}{c} = 7.44048\, {\frac{\mathrm{rad}}{\mathrm{s}^2}} \quad \Rightarrow \quad \varvec{\alpha }= \begin{bmatrix} -59.5238\\ 7.44048\\ 0 \end{bmatrix}\, {\frac{\mathrm{rad}}{\mathrm{s}^2}}. \end{aligned}$$

Alternatively, one can exploit that $\mathbf {a}_A$ is perpendicular to the tangent plane (actually, it is enough to use that $a_{Ax} = 0$), and

38 $$\begin{aligned} \mathbf {a}_B = \mathbf {a}_A + \varvec{\alpha }\times \mathbf {r}_{AB} + \varvec{\omega }_{}\times (\varvec{\omega }_{}\times \mathbf {r}_{AB}). \end{aligned}$$

With this approach, both the angular acceleration $\varvec{\alpha }$ and the acceleration of point *A* can be determined:

39 $$\begin{aligned} \mathbf {a}_{A} = \begin{bmatrix} 0\\0\\29.7619 \end{bmatrix} \, {\frac{\mathrm{m}}{\mathrm{s}^2}}, \end{aligned}$$

that corresponds to (33). The accelerations of $P_{g1}$ and $P_{g2}$ can be obtained similarly. Once the pole accelerations are known, the pole changing velocities can be determined by (13), leading to the same results as in (32).

Note that using formula (10), one obtains

40 $$\begin{aligned} {\tilde{\mathbf {u}}}_{P_{g2}} = \begin{bmatrix} 1.92\\0.58\dot{3}\\0 \end{bmatrix} \, {\frac{\mathrm{m}}{\mathrm{s}}}, \end{aligned}$$

due to the motion of the projection of point *D* parallel with the *y*-axis.

We can conclude that the accelerations of the points along the instantaneous axis of rotation could be determined somewhat easier using the pole velocities than by Euler’s formula. Moreover, some information was needed about the acceleration of a contact point (e.g., $\mathbf {a}_A$) or the angular acceleration $\varvec{\alpha }$ for the solution of the problem by Euler’s formula—this was the motivation of the present study.

A further advantage of the proposed solution is that while the apparent motion of the geometric pole can be described vividly, creating a mental picture about the acceleration vectors is more difficult.

### Rotation with slipping

Before relaxing the constraint of rolling, we analyze how this constraint influences the angular velocity and angular acceleration. As it was pointed out in the previous section, the magnitudes of the angular velocity of transportation and the relative angular velocity are not independent during rolling:

41 $$\begin{aligned} |\varvec{\omega }_t| = |\varvec{\omega }_r| \frac{r}{R}. \end{aligned}$$

The angular acceleration can be decomposed similarly, as shown in Fig. 9:

42 $$\begin{aligned} \varvec{\alpha }= \varvec{\alpha }_t + \varvec{\alpha }_r + \varvec{\omega }_t \times \varvec{\omega }, \end{aligned}$$

where $\varvec{\alpha }_t$ is the angular acceleration of transportation, $\varvec{\alpha }_r$ is the relative angular acceleration, while the term $\varvec{\omega }_t \times \varvec{\omega }$ is referred to as rotational angular acceleration in [26]. $\varvec{\alpha }_r$ and $\varvec{\alpha }_t$ characterize the change of the magnitude of $\varvec{\omega }_r$ and $\varvec{\omega }_t$, respectively. Consequently, it follows from (41) that the same relation must be fulfilled between the corresponding angular acceleration components:

43 $$\begin{aligned} |\varvec{\alpha }_t| = |\varvec{\alpha }_r| \frac{r}{R}. \end{aligned}$$

If this condition is fulfilled, the resultant $\varvec{\alpha }_\parallel \equiv \varvec{\alpha }_t +\varvec{\alpha }_r = \varvec{\alpha }-\varvec{\omega }_t \times \varvec{\omega }$ is always parallel with the angular velocity $\varvec{\omega }$ and the change of the direction of $\varvec{\omega }$ is characterized by the term $\varvec{\alpha }_\perp \equiv \varvec{\omega }_t \times \varvec{\omega }$. As illustrated in Fig. 9, the vectors $\varvec{\omega }$ and $\varvec{\alpha }_\perp$ span the tangent plane of the axodes.

If the ratio of $|\varvec{\alpha }_t|$ and $|\varvec{\alpha }_r|$ does not correspond to the rolling constraint, the ratio of the angular velocity components will change and the cone starts to slip.

**Fig. 10:**
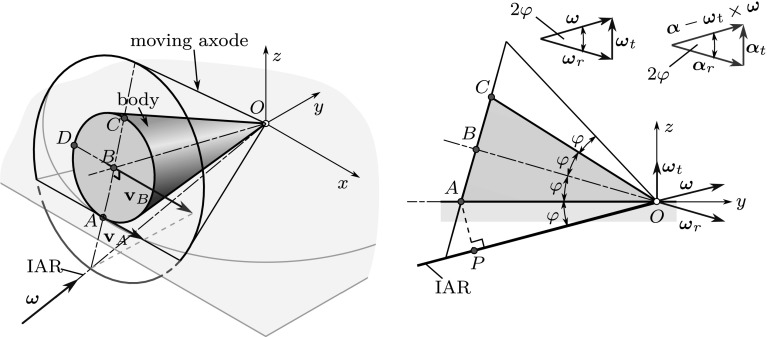
Illustration of the numerical example: sliding motion of a cone on a horizontal surface. The moving axode is another cone that does not coincide with the contour of the body. The IAR is just at the contact line of the moving axode and the fixed axode—the latter is not depicted in the figure

Figure 10 illustrates the situation when

44 $$\begin{aligned} \frac{|\varvec{\alpha }_t|}{|\varvec{\alpha }_r|} = \frac{|\varvec{\omega }_t|}{|\varvec{\omega }_r|} = 2 \frac{r}{R}. \end{aligned}$$

Now, these ratios define the geometry of another cone—the moving axode—with twice as large angle at the apex as the angle of the “material” cone. The fixed axode is another cone, below the moving axode that rolls on it without slip. Thus, this case can be treated similarly as the case of rolling in the previous subsection.

### Transition between the previous cases

Consider the case when $|\varvec{\alpha }-\varvec{\omega }_t\times \varvec{\omega }| = 10\, {{\mathrm{rad}}/{\mathrm{s}^2}}$ and

45 $$\begin{aligned} \frac{|\varvec{\alpha }_t|}{|\varvec{\alpha }_r|} = 2 \frac{r}{R}, \quad \text{ but }\quad \frac{|\varvec{\omega }_t|}{|\varvec{\omega }_r|} = \frac{r}{R}. \end{aligned}$$

Since these ratios are different, the components of $\varvec{\alpha }$ can be determined based on the vector triangle in Fig. 10, while the components of $\varvec{\omega }$ are related to each other as shown in Fig. 9. Initially, let the angular velocity be

46 $$\begin{aligned} \varvec{\omega }= \begin{bmatrix} 0\\\omega _{y}\\0 \end{bmatrix}= \begin{bmatrix} 0\\ 14.2857 \\0 \end{bmatrix}{\frac{\mathrm{rad}}{\mathrm{s}}}, \end{aligned}$$

as in Sect. 6.1. Assume that in this initial instant, point *A* is just on the IAR, as shown in Fig. 9.

According to the vector triangle in Fig. 10, the components of the angular acceleration are

47 $$\begin{aligned} \varvec{\alpha }= \begin{bmatrix} -|\varvec{\omega }_t \times \varvec{\omega }|\\ |\varvec{\alpha }-\varvec{\omega }_t\times \varvec{\omega }| \cos (\varphi )\\ 2|\varvec{\alpha }-\varvec{\omega }_t\times \varvec{\omega }| \sin (\varphi )\\ \end{bmatrix} = \begin{bmatrix} -59.5238\\ 9.6\\ 5.8 \end{bmatrix}\, {\frac{\mathrm{rad}}{\mathrm{s}^2}}. \end{aligned}$$

Since the ratio of angular acceleration components does not correspond to rolling, the direction of the angular velocity and the IAR starts to change. Within a finite time, the original cone of the moving axode (the smaller cone in Fig. 10) is transformed into the larger cone. It is rather difficult to imagine how the moving and fixed axodes are transformed during the motion, even the position of the common tangent plane of the axodes is hard to see.

To determine the tangent plane, one can exploit that the pole changing velocity and the angular velocity span this plane. For the calculation of $\mathbf {u}$, Eq. (13) can be utilized.

Using Euler’s formula, the acceleration of point *A* can be determined:

48 $$\begin{aligned} \mathbf {a}_A = \varvec{\alpha }\times \mathbf {r}_{OA} + \varvec{\omega }\times (\varvec{\omega }\times \mathbf {r}_{OA}) = \begin{bmatrix} 2.9\\0\\ 29.7619 \end{bmatrix}\, {\frac{\mathrm{m}}{\mathrm{s}^2}}. \end{aligned}$$

According to (13), the pole changing velocity of the geometric pole coinciding *A* is

49 $$\begin{aligned} \mathbf {u}= \frac{\varvec{\omega }\times \mathbf {a}_A}{\omega ^2} = \begin{bmatrix} 2.08\dot{3}\\0\\ -0.203 \end{bmatrix}\, {\frac{\mathrm{m}}{\mathrm{s}}}. \end{aligned}$$

The components of this vector can be determined in an alternative way, too. The *x* component of the pole changing velocity can be calculated by taking into account the transportation component $\varvec{\omega }_t$ of the angular velocity:

50 $$\begin{aligned} u_x = R \varvec{\omega }_t = 2.08\dot{3}\,\mathrm{m/s}. \end{aligned}$$

To determine the other component, we can exploit that in the case of spherical motions,

51 $$\begin{aligned} \varvec{\alpha }_\perp \equiv \varvec{\alpha }-\frac{(\varvec{\alpha }\cdot \varvec{\omega }) \varvec{\omega }}{\omega ^2} = \begin{bmatrix} -59.5238\\0\\ 5.8 \end{bmatrix}\, {\frac{\mathrm{rad}}{\mathrm{s}^2}} \end{aligned}$$

is perpendicular to $\varvec{\omega }$ and is parallel to the tangent plane—just like $\mathbf {u}$. Thus, $\varvec{\alpha }_\perp$ is parallel with $\mathbf {u}$, that implies that

52 $$\begin{aligned} u_z =\frac{u_x}{\alpha _x}\alpha _z = -0.035 \cdot 5.8 = -0.203. \end{aligned}$$

Thus,

53 $$\begin{aligned} \mathbf {u}= \begin{bmatrix} 2.08\dot{3}\\0\\ -0.203 \end{bmatrix}\, {\frac{\mathrm{m}}{\mathrm{s}}} \end{aligned}$$

and using (17), the pole acceleration can be expressed, too:

54 $$\begin{aligned} \mathbf {a}_A = \mathbf {u}\times \varvec{\omega }= \begin{bmatrix} 2.9\\0\\ 29.7619 \end{bmatrix}\, {\frac{\mathrm{m}}{\mathrm{s}^2}}. \end{aligned}$$

In this problem, the direction of the tangent plane of the axodes cannot be determined without performing the calculations. Still, if the angular acceleration is known, the magnitude and direction of $\mathbf {u}$ can be expressed and the pole acceleration can be calculated quite easily.

## Conclusions

The present contribution showed a generalization of the concept of pole changing velocity $\mathbf {u}$ to general spatial motions of rigid bodies. It was pointed out using Euler’s rigid body formulas that the pole changing velocity can be defined in such a way that it becomes independent of the reference point:

55 $$\begin{aligned} \mathbf {u}= \mathbf {v}_P + \frac{\varvec{\omega }\times \mathbf {a}_P}{\omega ^2}, \end{aligned}$$

where $$\mathbf {v}_P \parallel \varvec{\omega }$$ (Eq. 12).

This formula is almost the same as the corresponding formula in planar kinematics. The only difference is the appearance of the pole velocity $\mathbf {v}_P$ (the velocity of the material point on the ISA) that is nonzero only in the case of raccording (or screw) motion. As a consequence, the pole acceleration $\mathbf {a}_P$ can be expressed in the form

56 $$\begin{aligned} \mathbf {a}_{P} = \mathbf {u}\times \varvec{\omega }, \end{aligned}$$

provided that the body exhibits rotational motion [see (18)]. In addition to these formulas, connections among the directions of vectors $\mathbf {u}$, $\varvec{\omega }$, $\varvec{\alpha }$ and $\mathbf {a}_P$ were also derived.

Since the direction of the pole changing velocity is parallel with the common tangent of the moving and fixed axodes, its direction and magnitude can be often determined easily. Thus, the obtained results can be utilized for the quick derivation of the acceleration of a chosen material point on the ISA or for the check of calculations based on other methods.

In principle, the results of the paper can be derived as special cases of more general geometrical results. Still, the author did not find these statements written explicitly in the literature. Thus, the goal of the presented calculations is to help to comprehend and visualize the spatial motions of rigid bodies.

## Footnotes

1 It is worth to compare this result with (10).
